# Supplementary material for: Outcomes of Extracorporeal Membrane Oxygenation for Acute Respiratory Distress Syndrome in COVID-19 Patients: A Propensity-Matched Analysis
Source: J Clin Med. 2021 Jun 9;10(12):2547. doi: 10.3390/jcm10122547 (PMC8227073; doi:10.3390/jcm10122547)
Supplement: Supplementary file 1 [file jcm-10-02547-s001.zip › jcm-1234237-supplementary.pdf]

Supplementary Table S1. Characteristics of the whole cohort prior to matching.

	COVID (n=20)	NCOVID (n=105)	p-value
**Age (y)**	57 (50-62)	54 (42-61)	0.182
**Female gender, n (%)**	6 (30)	37 (35)	1.000
**Weight (kg)**	90 (80-100)	82 (71-100)	0.329
**Height (cm)**	176 (170-185)	175 (165-181)	0.344
**BMI (kg/m^2)**	28.2 (24.7-31.1)	27 (25-33)	0.617
**Pre-ECMO LOS in hospital (d)**	6 (4-16)	0 (0-1)	0.000*
**Coronary Artery Disease, n (%)**	1 (5)	16 (15)	0.467
**Prior Myocardial Infarction, n (%)**	1 (5)	11 (10)	0.690
**VA-ECMO n(%)**	1 (5)	38 (33)	0.007
**Arterial Hypertension, n (%)**	14 (70)	35 (33)	0.002*
**COPD, n (%)**	3 (15)	17 (16)	1.000
**Diabetes mellitus type 2, n (%)**	7 (35)	16 (15)	0.048
**Pulmonary Hypertension, n (%)**	2 (10)	0 (0)	0.022*
**Chronic kidney disease, n (%)†**	2 (10)	10 (10)	0.000*
**Nicotine use, n (%)**	4 (20)	43 (41)	0.126
**Immunosuppressive medication, n (%)**	1 (5)	16 (15)	0.467
**History of Malignancy, n (%)**	1 (5)	5 (5)	1.000
**Laboratory parameters**			
**pO2 (mmHg)**	68 (54-72)	77 (60-116.5)	0.023*
**FiO2 (percentage)**	60 (50-80)	82 (54-100)	0.036*
**Creatinine (mg/dL)**	1.2 (0.9–1.7)	1.07 (0.76-1.86)	0.091
**Bilirubin (mg/dL)**	0.6 (0.45–1.9)	0.56 (0.32-1.8)	0.054
**Hemoglobin (g/dL)**	9.8 (8.8–10.5)	10.5 (9.375-11.5)	0.105
**Respiratory Extracorporeal Membrane Oxygenation Survival Prediction Score**			
**Total RESP Score**	0 (-3-2)	2 (1-5)	0.003*
**Survival Prediction (percentage)**	57 (33-57)	57 (57-76)	0.001*
**$PaCO_2$ ≥75 mmHg, n (%)**	6 (32)	23 (22)	1.000
**Peak inspiratory pressure ≥42cmH_2O, n (%)**	0 (0)	1 (1)	1.000
**Outcomes**			
**90-day mortality, n (%)†**	9 (45)	61 (58)	0.455
**ECMO duration (d)**	16 (11-23)	12 (7-20)	0.129
**Total LOS in hospital (d)**	29 (14-55)	29 (11-53)	0.638
**Thromboembolic Events, n (%)**	8 (40)	18 (17)	0.028*
**Pulmonary Artery Embolism**	6 (30)	5 (5)	0.008*
**Peripheral Venous Thrombosis**	4 (20)	8 (8)	0.121
**Peripheral Arterial Thrombosis**	1 (5)	5 (5)	1.000
**Other Thromboembolic Events††**	0 (0)	5 (5)	1.000
**Major Bleeding Events, n (%)**	9 (45)	44 (42)	1.000
**Endobronchial**	2 (11)	18 (17)	0.736
**Mucosal**	3 (16)	6 (6)	0.141
**Cannulation Side**	2 (10)	19 (18)	0.526
**Gastrointestinal**	2 (10)	2 (2)	0.119
**Cerebral**	0 (0)	4 (4)	1.000
**Hemothorax**	1 (5)	14 (13)	1.000

Continuous variables are presented as median and interquartile range (IQR). *p values under 0.05 are significant and tagged with asterisks. Abbreviations: BMI—body mass index kg/m2; ECMO—extracorporeal membrane oxygenation; ICU—intensive care unit; LOS—length of stay; NCOVID—non-COVID; VV—venovenous; †Death during 90 days after initiation of ECMO therapy.

Supplementary Table S2. Laboratory parameters.

	COVID (n=20)	NCOVID (n=34)	p-value
**Hb (g/dl)**			
Pre-ECMO	9.8 (8.8-10.5)	9.6 (8.9-11.4)	0.764
24h after ECMO-initiation	9.6 (8.8-10.7)	9.8 (8.9-11)	0.536
Pre-Explantation	9.7 (9.3-10.5)	9.6 (8.2-10.7)	0.447
**Leucocytes (/nL)**			
Pre-ECMO	13 (10-17)	11 (9-19)	0.558
24h after ECMO-initiation	14 (10-19)	11.4 (7-14)	0.122
Pre-Explantation	13 (8-18)	12 (6-14)	0.294
**Platelet (G/L)**			
Pre-ECMO	222 (165-395)	131 (87-214)	0.074
24h after ECMO-initiation	205 (124-304)	106 (38-171)	0.05*
Pre-Explantation	163 (96-208)	86 (54-120)	0.02*
**PCT (percentage)**			
Pre-ECMO	4 (2-6)	1 (0-6)	0.053
24h after ECMO-initiation	4 (2-7)	2 (1-13)	0.266
Pre-Explantation	6 (2-10)	2 (1-6)	0.054
**aPTT (s)**			
Pre-ECMO	30 (29-45)	32 (29-53)	0.174
24h after ECMO-initiation	33 (29-40)	41 (31-52)	0.036*
Pre-Explantation	41 (29-50)	39 (31-52)	0.767
**INR (ratio)**			
Pre-ECMO	1.3 (1.2-1.4)	1.1 (1-1.2)	0.899
24h after ECMO-initiation	1.2 (1.1-1.4)	1.2 (1.1-1.2)	0.334
Pre-Explantation	1.3 (1.2-1.7)	1.2 (1.1-1.4)	0.253
**Fibrinogen (mg/dL)**			
Pre-ECMO	493 (454-704)	364 (276-490)	0.000*
24h after ECMO-initiation	455 (322-470)	369 (308-475)	0.324
Pre-Explantation	268 (223-414)	329 (256-386)	0.564
**D-dimer (µg/dL)**			
Pre-ECMO	4345 (1810-11806)	2400 (1782-7030)	0.253
24h after ECMO-initiation	7591 (3878-15030)	3754 (2200-10000)	0.065
Pre-Explantation	24689 (8306-62398)	3860 (2041-9658)	0.011*
**ATIII (%)**			
Pre-ECMO	70 (57-78)	58 (40-75)	0.155
24h after ECMO-initiation	53 (45-66)	64 (48-77)	0.074
Pre-Explantation	79 (66-89)	67 (56-90)	0.280
**pfHb (mg/L)**			
Pre-ECMO	36 (22-50)	48 (30-72)	0.162
24h after ECMO-initiation	45 (35-70)	68 (44-138)	0.041*

Pre-Explantation	80 (62-122)	120 (36-305)	0.775
**LDH (U/L)**			
Pre-ECMO	403 (312-578)	466 (318-644)	0.589
24h after ECMO-initiation	480 (405-560)	515 (321-765)	0.937
Pre-Explantation	513 (432-756)	518 (344-1032)	0.578
**AST (U/L)**			
Pre-ECMO	66 (38-140)	62 (32-164)	0.967
24h after ECMO-initiation	65 (48-142)	59 (29-196)	0.900
Pre-Explantation	86 (46-165)	88 (42-242)	0.831

All variables are presented as median and interquartile range (IQR). *p values under 0.05 are significant and tagged with asterisks. Abbreviations: AST—aspartate transaminase; aPTT—partial thromboplastin time; ECMO—extracorporeal membrane oxygenation; fHb—free-plasma hemoglobin; Hb—hemoglobin; LDH—lactate dehydrogenase; LOS—length of stay; NCOVID—non-COVID; PCT—procalcitonin.

Figure S1. Standardized absolute bias difference pre and post matching.

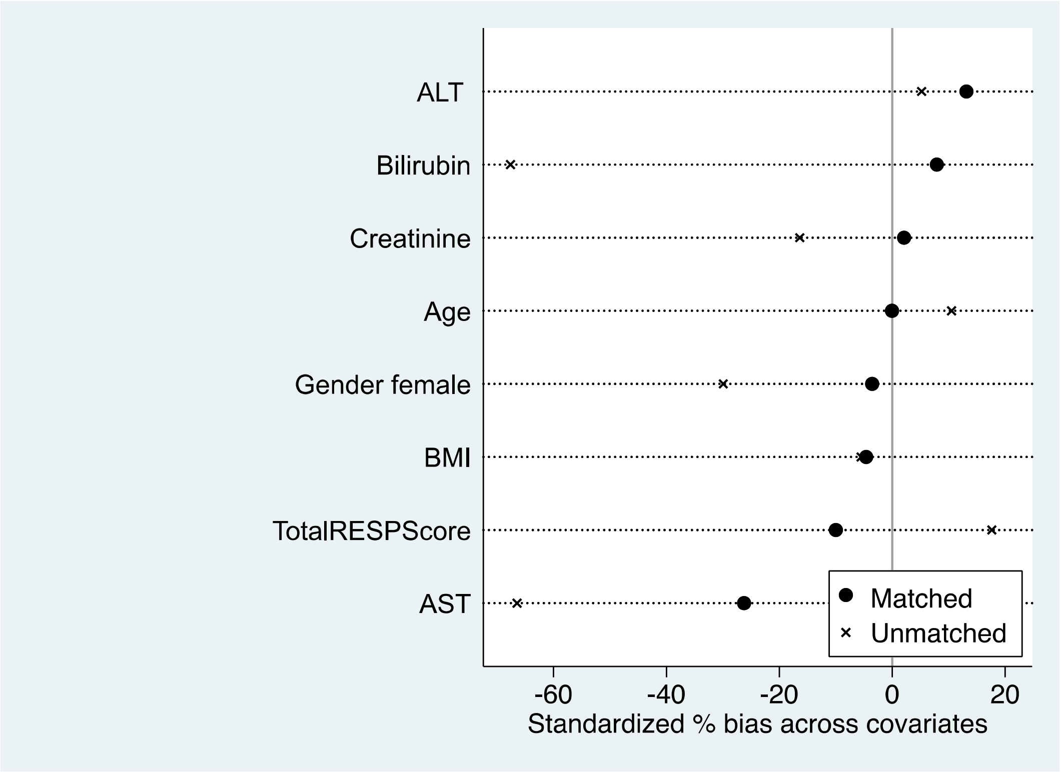

ALT—alanine aminotransferase U/L; AST—aspartate aminotransferase U/L; BMI—body mass index Kg/m^2.

Figure S2. Comparison of the time course of activated partial thromboplastin time (aPTT) between the COVID group and the NCOVID group.

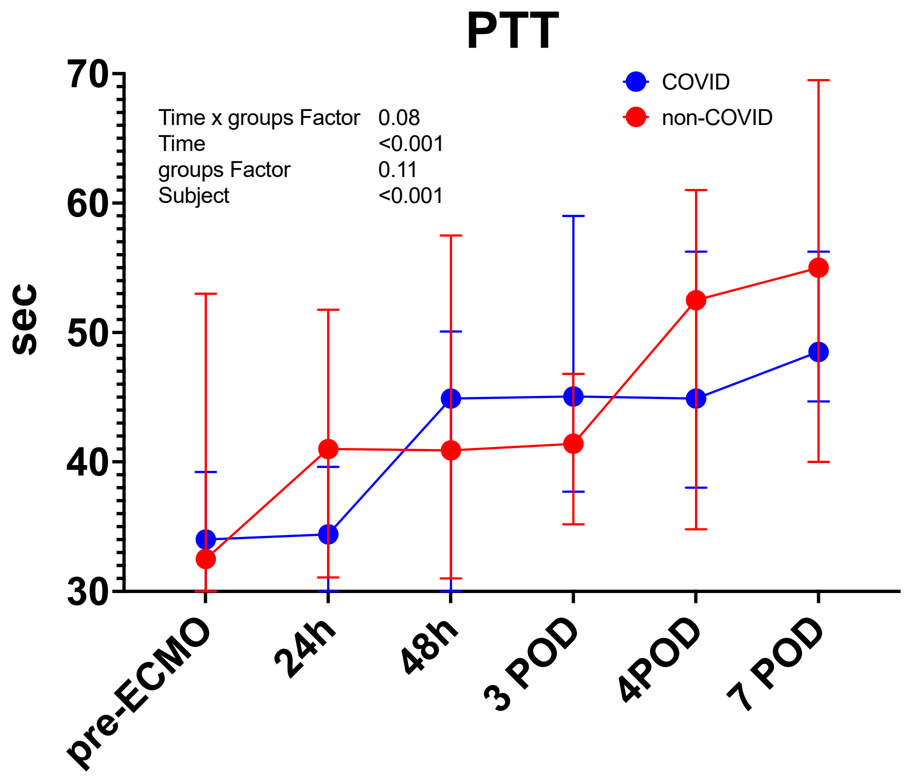

Comparison was performed with two way-ANOVA with adjusted *p*-values for repeated measurements. aPTT did not differ significantly between the two groups.
